# 

**Published:** 1875-05-15

**Authors:** 


*ORIGINAL COMMUNICATIONS:*

Irrigation in a Recent Wound. By Chas. Hervey Hunt, M.D., ........ 267

A Case of Hernia Proven by Post-Mortem to Contain Bile and Pus. By U. P. Stair, M.D., 268

Quadruplets. Notes from Practice of J. J. Schneck, M. D., ........ 269

*TRANSLATIONS:*

Clinical Thermometry. By Dr. B. Kraus. Condensed and Translated by Dr. H. Gradle. Continued. ........ 270

Gleanings from the German. By Dr. H. Gradle, 274

*EDITORIAL DEPARTMENT:*

American Medical Association........ 277

Association of American Medical Editors........ 280

*GLEANINGS FROM OUR EXCHANGES:*

Correspondence........ 281

Dermatological........ 282

Discussion of Tumors by Electricity........ 284

Changes in the New York Schools........ 285

*BOOK REVIEWS* ........ 292

